# Compliance with adjuvant capecitabine in patients with stage II and III colon cancer: comparison of administrative versus medical record data

**DOI:** 10.1002/cam4.745

**Published:** 2016-05-26

**Authors:** Adam Amlani, Aalok Kumar, Jenny Y. Ruan, Winson Y. Cheung

**Affiliations:** ^1^ Division of Medical OncologyBritish Columbia Cancer AgencyVancouverBritish ColumbiaCanada

**Keywords:** Adjuvant, cancer, capecitabine, colon, compliance

## Abstract

We aimed to examine the frequency of treatment delays as well as the reasons and appropriateness of such delays in early stage colon cancer patients receiving adjuvant capecitabine by comparing data from pharmacy dispensing versus medical records. Patients diagnosed with stage II or III colon cancer from 2008 to 2012 and who received at least two cycle of adjuvant capecitabine were reviewed for treatment delays. Data from pharmacy dispensing and patient medical records were compared. Multivariate regression models were constructed to identify predictors of treatment delays. A total of 697 patients were analyzed: median age was 70 years (IQR 30–89), 394 (57%) were men, 598 (86%) reported Eastern Cooperative Oncology Group 0/1, and 191 (27%) had stage II disease. In this study cohort, 396 (57%) patients experienced at least 1 treatment delay during their adjuvant treatment. Upon medical record review, half of treatment delays identified using pharmacy administrative data were actually attributable to side effects, of which over 90% were considered clinically appropriate for patients to withhold rather than to continue the drug. The most prevalent side effects were hand‐foot syndrome and diarrhea which occurred in 176 (44%) and 67 (17%) patients, respectively. Multivariate analysis revealed a statistically significant association between stage and inappropriate treatment delays whereby patients with stage II disease were more likely to experience drug noncompliance (OR 1.79, 95% CI: 1.27–2.53, *P* < 0.001) than those with stage III disease. Compliance with adjuvant capecitabine was reasonable. Adherence ascertained from pharmacy administrative data differs significantly from that obtained from medical records.

## Introduction

The number of anticancer therapies available in oral formulation has increased significantly over the past decade 1, 2. While oral agents pose many notable advantages when compared to conventional parenteral drugs, including enhanced convenience of self‐administration, reduced hospital and societal costs, and improved patient engagement in their own care 1, 2, 3, 4, these benefits must be balanced against growing concerns regarding poor compliance to treatment and its potentially negative impact on outcomes 1, 2, 3, 4, 5, 6. Suboptimal adherence to medications is a commonly recognized problem for many chronic medical conditions, such as hypertension and diabetes mellitus, where it has been shown to compromise drug effectiveness 7, 8, 9, 10.

The increased availability and use of oral anticancer agents have prompted a growing level of attention toward medication adherence among cancer patients. The majority of studies on medication compliance in oncology have focused on oral hormonal therapies in breast cancer 5, 6, 11, 12, 13 even though oral drugs are also frequently used in other cancers 14, 15, 16, 17. In colon cancer, the oral 5‐fluorouracil prodrug known as capecitabine is a commonly prescribed component in a large number of systemic therapy regimens 18, 19, but it has received relatively little focus to date 20, 21, 22, 23. Patients prescribed capecitabine may be at risk of not filling the initial prescription (noninitiation), failing to take the medication as prescribed (nonadherence), or continuing with the drug only temporarily (early discontinuation or nonpersistence) 20, 22.

Cancer patients are particularly vulnerable to oral drug noncompliance because many are elderly and predisposed to polypharmacy due to the need to take concomitant medications to manage other comorbidities 24, 25. Compliance is further hindered by systemic therapy drugs that often have significant toxicities or complicated dosing schedules (e.g., 2 weeks on and 1 week off) 26, 27, 28. The latter poses significant challenges for researchers because conventional metrics used to measure noncompliance, such as proportion of days covered (PDC), cannot always be readily applied when drugs are administered by cycles rather than on a daily basis or a fixed schedule 20, 29. Instead of PDC, it may be more reasonable to evaluate delays in chemotherapy prescriptions as a proxy of compliance 30. In oncology, there are also valid clinical indications such as toxicities whereby it may be more appropriate for patients to withhold rather than to continue a drug 29. Thus, the conventional methodology of relying on pharmacy dispensing records alone to study drug compliance may potentially lead to misclassification or unreliable ascertainment of adherence patterns.

Given these challenges, our aims were to 1) examine oral drug compliance with capecitabine in the adjuvant treatment setting for colon cancer by assessing the frequency of prescription refill delays; 2) compare findings from pharmacy administrative data with those obtained from electronic medical records of patients; and 3) identify clinical factors and reasons associated with inappropriate noncompliance. We hypothesized that compliance with capecitabine is reasonable in the general ‘real world’ population and that information from clinical records provides a more accurate assessment of chemotherapy adherence when compared to pharmacy dispensing data alone.

## Methods

### Description of the study setting

The British Columbia Cancer Agency is a province‐wide cancer control program that is responsible for providing publicly funded population‐based cancer treatment to approximately 5 million residents of British Columbia, Canada. During the study time period, the agency was composed of five regional cancer centers that were geographically distributed across different catchment areas of the province to ensure equitable access to cancer care for all of its residents. Each center offers a full range of oncological care, including ambulatory clinics, systemic therapy suites, radiation and surgical facilities, pain and symptom management, palliative care, and inpatient units for patients diagnosed with cancer. A provincial pharmacy with affiliated satellite sites at each of the five regional cancer centers is responsible for dispensing systemic therapy to all patients. In addition, patients are given opportunities to participate in oncology clinical trials. An estimated 15,000 to 20,000 new patients are referred to the British Columbia Cancer Agency annually for management. This study was conducted after receiving full research ethics approval from the institutional review board.

### Description of the patient population

We included all patients who were diagnosed with either stage II or III colon cancer from 2008 to 2012, referred to any one of the five comprehensive cancer centers of the British Columbia Cancer Agency for management, and received at least two cycles of any adjuvant intent capecitabine chemotherapy with the requirement that the first cycle was administered within 12 weeks after curative resection. This specific 5‐year study time period was selected in order to allow for adequate sample size and sufficient follow‐up for reliable ascertainment of outcomes. At our institution, all systemic therapy regimens are coded to reflect treatment intent (e.g., adjuvant vs. palliative), thus allowing this analysis to focus specifically on a cohort of early stage colon cancer patients treated with adjuvant capecitabine and in whom noncompliance may have a greater impact on the likelihood of cure.

### Ascertainment of capecitabine compliance

Capecitabine is administered at a dose of 1250 mg/m^2^ orally every 12 h on days 1 to 14 of each 21 day cycle. Therefore, compliance to capecitabine was assessed by ascertaining whether receipt of a subsequent chemotherapy cycle occurred at the expected 21‐day interval following the initiation of the preceding cycle. Noncompliance was defined as any delays of ≥7 days from the anticipated date of delivery of the next course of capecitabine chemotherapy. This definition was selected in order to minimize misclassification of patients who may have experienced minor schedule deviations of a few days in order to accommodate for delays secondary to statutory holidays or brief negotiated absences for personal or professional reasons. As a sensitivity analysis, we also modified our definition of noncompliance by using delays of either over 4 or over 10 days. Because results did not differ significantly from the primary analysis, only the main findings are presented.

Compliance was initially evaluated using pharmacy administrative data that capture the dates of oral pill dispensing. This was subsequently corroborated with electronic medical records containing patients’ clinical and treatment history. In cases of discrepancies whereby pills were dispensed, but patients reported that the drugs were taken neither as prescribed nor on time, this information would appear solely in the electronic medical records and thus it would be used as the preferred data source. Clinical charts were further reviewed to ascertain reasons for treatment delays and subsequently categorized as appropriate versus inappropriate clinical indications by two investigators (AA, JYR) who were blinded from each other. Discordant interpretations between the two investigators were 3% and disagreements were resolved through consensus by involving the senior investigator (WYC) who conducted a separate review.

### Definitions of covariates

In addition to the frequency, duration, and reasons for prescription delays, further patient information was collected from electronic medical records in order to evaluate individual factors associated with noncompliance. This included patient demographics (e.g., age, gender, Eastern Cooperative Oncology Group [ECOG] status prior to treatment with capecitabine) as well as disease characteristics (e.g., stage). Concurrent comorbidities, concomitant prescription medications, and toxicities were also collated and categorized.

### Statistical analysis

Patient‐ and treatment‐level characteristics for the study cohort were summarized with descriptive statistics. Differences in baseline features between treatment compliant and noncompliant patients were evaluated using the Mann–Whitney test for continuous variables and the Chi‐Square test for categorical variables. We compared data between pharmacy dispensing records and electronic medical records using concordance rates. Multivariate logistic regression models that adjusted for confounders, such as age, gender, and baseline ECOG, were subsequently constructed to identify potential predictors of treatment delays that were considered clinically inappropriate. All tests were two‐sided where a *P*‐value of <0.05 was considered statistically significant. The R statistical package was used for all of the statistical analyses.

## Results

### Patient‐ and treatment‐level characteristics

In total, we identified 697 eligible patients for this study. Median age was 70 years (IQR 30–89), 394 (57%) were men, 598 (86%) reported ECOG 0/1, and 191 (27%) had stage II disease. In this cohort, 396 (57%) of patients experienced at least one treatment delay during their course of adjuvant capecitabine chemotherapy according to both pharmacy administrative data and medical record review. Concordance between the two data sources with respect to whether or not a delay occurred was 97%. There were no significant differences in baseline characteristics between treatment compliant and noncompliant individuals in terms of age, gender, baseline ECOG, comorbidities, and number of concomitant medications (all *P* > 0.05). However, when compared to patients who were compliant, those who were noncompliant were more likely to have been diagnosed with stage II rather than stage III colon cancer (66% vs. 78%, *P* < 0.0001). Additional details are highlighted in Table 1.

**Table 1:** Baseline Characteristics of Study Cohort

Variable	Total *n* = 697 (%)	No Treatment Delay		Treatment Delay		*P*‐value
		*n* = 301	%	*n* = 301	%	
Age						0.98
Range	30–89	30–89		35–87		
Median	70	70		70		
<70 years	362 (52)	157	43	205	57	
70 years or older	335 (48)	144	43	191	57	
Gender						0.96
Female	303 (43)	130	43	173	57	
Male	394 (57)	171	43	223	57	
Performance Status^a^						0.21
0	287 (41)	137	48	150	52	
1	311 (45)	122	39	189	61	
2	80 (11)	34	43	46	57	
3	18 (3)	7	39	11	61	
Comorbidities^b^						0.37
0–1	130 (19)	61	47	69	53	
2 or more	565 (81)	238	42	327	58	
Number of Medications^a^						0.20
3 or Less	398 (57)	181	45	217	55	
4 or More	298 (43)	119	40	179	60	
Stage						<0.00001
2	191 (27)	103	54	88	46	
3	506 (73)	198	39	308	61	

Except for the total column, all percentages are row percentages.

a Data missing on one patient.

b Data missing on two patients.

Among the 697 patients, a total of 4097 chemotherapy cycles were administered during the study time period of which 657 (16%) cycles were delayed. Cycle‐level characteristics are summarized in Table 2. While the majority of patients (N 219; 55%) who were noncompliant filled their capecitabine prescription late on only one occasion, the remainder (N 177; 45%) experienced two or more delays. The median treatment delay was 28 days (IQR 25–144).

**Table 2:** Delays in planned oral capecitabine therapy

Patients Who Were Delayed		
*Duration of Delay (days)*		
Range	25–144
Median	28
Mean	31.8
*Frequency of Delays*		
1 delay/patient	219 (55)
>1 delay/patient	177 (45)	
Reasons For Delays Based on Medical Records	Total	Clinically Appropriate
Side Effects	329	326
Physician	158	155
Patient	21	4
Travel	40	37
Other/Unknown	80	3

### Clinical indications for delays

Comprehensive review of the electronic medical records demonstrated that most (84%) of the treatment delays were intentional and due to the following common reasons: toxicities (62%), physician discretion not otherwise specified (30%), logistics of clinic and treatment scheduling (7%), personal or professional reasons (e.g., extended leisure or business travel) (1%), or patient preference and request to hold therapy (1%). For each of these categories except for patient preference, over 90% of cases were considered clinically appropriate for the patient to withhold rather than to continue the drug. In cases of patient preference, however, the majority was perceived as clinically inappropriate to stop therapy. Among the 697 cycle delays, only 96 (15%) were considered clinically inappropriate treatment delays and thus represented *true* drug noncompliance. Common side effects that contributed to appropriate treatment delays included hand‐foot syndrome (44%), diarrhea (17%), and other skin toxicities (8%), which are comparable to those observed in clinical trials 31. In our cohort, there were also some cases of extended treatment delays that persisted for several months. Reasons included prolonged hospital admissions secondary to surgical procedures, postoperative complications, or severe toxicities as well as patient request to temporarily stop therapy due to travel. For the subgroup of ECOG three patients, the reasons for treatment delays were primarily side effects. Review of these patients’ medical charts showed that these individuals also suffered poor medical fitness which may have played a significant role in their poor tolerance and inability to recover from chemotherapy side effects.

### Predictors of inappropriate treatment delays

On multivariate analysis (Table 3) that adjusted for known confounders, we observed a statistically significant association between stage and inappropriate treatment delays whereby those with stage II disease were more likely experience drug noncompliance (OR 1.79, 95% CI: 1.27–2.53, *P* < 0.001). Of note, demographic characteristics such as advanced age (OR 1.00, 95% CI: 0.98–1.01, *P* = 0.71) and gender (OR 0.97, 95% CI: 0.72–1.32, *P* = 0.87) did not correlate with delays. Likewise, ECOG performance status, number of comorbidities, and number of concomitant medications did not predict for treatment noncompliance (OR 0.97, 95% CI: 0.62–1.51, *P* = 0.88; OR 1.17, 95% CI: 0.84–1.64, *P* = 0.35; OR 1.13, 95% CI: 0.80–1.58, *P* = 0.49, respectively).

**Table 3:** Multivariate logistic regression predicting for delay

Variable	OR	95% CI	*P*‐value
Age	1.00	0.98–1.01	0. 71
Gender			
Male vs. Female	0.97	0.72–1.32	0.87
Performance Status			
2–3 vs. 0–1	0.97	0.62–1.51	0.88
Comorbidities			
2 or more vs. 0–1	1.17	0.84–1.64	0.35
Number of Concomitant Medications			
4 or more vs. 3 or less	1.13	0.80–1.58	0.49
Stage			
3 vs 2	1.79	1.27–2.53	<0.001

Age analyzed as a continuous variable. When analyzed as categorical, no significant different found with the model.

## Discussion

Compliance with oral anticancer therapies is infrequently studied in tumors other than breast cancer, but poor adherence to drugs is an increasingly pervasive problem in oncology as more agents are introduced in oral form. In this population‐based study of early stage colon cancer patients who were prescribed adjuvant capecitabine, patterns ascertained by pharmacy dispensing data significantly overestimated the rate of noncompliance. We found that the majority of treatment delays were clinically warranted due to toxicities and that only a minority of cases represented clinically inappropriate treatment interruptions or true drug noncompliance. Future studies evaluating oral drug compliance in oncology should be cognizant of the limitations of relying solely on administrative data. Ideally, investigators should leverage the strengths of clinical information available in medical records to help discern between appropriate and inappropriate adherence to treatment.

Importantly, most prior studies in the noncancer population utilized PDC as the metric of evaluation of drug adherence 32, 33, 34. This approach was also frequently used in analyses involving oral hormonal therapies in breast cancer 35, 36, 37. PDC provides reliable information when a drug is administered on a daily basis since PDC is derived by determining the percentage of days over a defined time period in which the drug was dispensed. While relatively straightforward to derive, PDC has inherent limitations 20, 29. In settings where a drug is administered periodically or at different doses or timing throughout the treatment course, PDC may not account for the complexities of these schedules and thus result in less reliable capture of compliance 20, 29. For adjuvant capecitabine in colon cancer, a “2 weeks on and 1 week off” schedule is employed so characterizing treatment delays may offer a more accurate impression of compliance. A patient who takes 4 weeks of capecitabine consecutively over a 6 week period, for instance, would produce a normal PDC even though this is inconsistent with the way that adjuvant capecitabine should ideally be delivered.

In general, we noted relatively strong adherence to adjuvant capecitabine since the frequency of inappropriate treatment delays was low at 15%. This is consistent with prior studies of oral cytotoxic chemotherapies 25 where the severity of a malignant diagnosis and its potential to lead to significant morbidity and mortality may cause patients to comply with medical advice, including taking their medications as prescribed. This is further supported by our observation that groups conventionally expected to be relatively noncompliant, such as patients with advanced age and polypharmacy 25, did not actually experience higher rates of treatment delays when compared to their counterparts. Interestingly, individuals with stage II disease were more likely than those with stage III disease to be noncompliant. One potential explanation is that the relatively small magnitude of benefit achieved with adjuvant capecitabine in stage II disease 38 exposes patients to a different trade‐off scenario whereby the risks of toxicities associated with continuing chemotherapy outweigh the potential survival advantages.

One of the distinguishing features of this study is our comparison of findings based on pharmacy administrative data versus those obtained from patient records, which allowed us to validate if pharmacy dispensing records were a reliable data source for ascertaining drug compliance. While there are a number of potential strategies for evaluating drug patterns, including biological testing (e.g., measuring serum and urine drug or metabolite levels) and self‐monitoring systems (e.g., surveys, pill counts, patient interviews) 20, pharmacy administrative data are most frequently used because they generally provide a large sample of patients on a specific drug 20. In addition, it avoids the Hawthorne effect in which patients may modify or improve their compliance for social desirability when they are aware that they are being evaluated 18. Furthermore, analysis of dispensing data is less expensive and laborious when compared with other study methods 20. Importantly, our current study underscores that pharmacy data can be potentially misleading and that review of medical records provides important granular information about the appropriateness of noncompliance. Because patient medical record review can be labor intensive, it may not always be feasible to conduct for a large sample of patients. However, corroborating even a small proportion of pharmacy data with clinical chart review may add significant context and validity to findings.

The results from this study should be interpreted in the context of several limitations. First, we focused on a specific population of early stage colon cancer patients who underwent treatment with adjuvant capecitabine, so our conclusions cannot be generalized to other tumor groups or individuals receiving other forms of systemic therapy. For instance, due to database limitations, we cannot comment on compliance with capecitabine when it is administered as part of a combination regimen, but we suspect that adherence may be lower when its delivery is complicated by the receipt of additional systemic agents such as oxaliplatin. Likewise, our results may not be applicable to young patients affected with aggressive disease in whom combination chemotherapy may be used preferentially over capecitabine monotherapy. Second, while we were able to capture delays as a measure of compliance, it was beyond the scope of this study to ascertain deviations from optimal dose intensity, largely because dose modifications can be secondary to a number of reasons, including patient preference, physician discretion, weight changes, and toxicities. Because we focused on adjuvant treatments where the aim is cure, we believe the likelihood that clinicians would make significant dose reductions is relatively low in most circumstances since there is evidence to suggest that cumulative dose intensity correlates with survival. Finally, given the retrospective nature of this study, we were unable to grade the severity of toxicities. However, these limitations must be considered against the study's strengths, including its focus on medical reasons of delays since universal health coverage of chemotherapy costs excludes any financial causes for delays.

In summary, compliance to capecitabine in this study cohort was generally fair. Adherence was worse in stage II colon cancer patients in whom adjuvant capecitabine is expected to have a smaller magnitude of benefit. This may highlight areas in need of improvement in patient‐physician communication during treatment decision making. Clinicians and researchers should exercise caution in solely using pharmacy dispensing data to estimate nonadherence, as this may include clinically appropriate treatment delays and thus significantly overestimate true noncompliance. If possible, corroborating findings with a complementary chart review may facilitate a more reliable ascertainment of drug adherence patterns.

## Conflicts of Interest

None declared.

## Supporting information


**Table S1.** Side Effects Summary.
