# Supplementary material for: Compliance with adjuvant capecitabine in patients with stage II and III colon cancer: comparison of administrative versus medical record data
Source: Cancer Med. 2016 May 26;5(8):1776–82. doi: 10.1002/cam4.745 (PMC4884630; doi:10.1002/cam4.745)
Supplement: Supplementary file 1 — Table S1. Side Effects Summary. [file CAM4-5-1776-s001.docx]

Table (Online Only): Side Effects Summary

| **Side Effects** | **Frequency** |
| --- | --- |
| **None** | 86 |
| **HFS** | 176 |
| **Diarrhea** | 67 |
| **Skin** | 30 |
| **Mucositis** | 28 |
| **Fatigue** | 32 |
| **Nausea & Vomiting** | 19 |
| **Pain** | 18 |
| **Low ANC / Infection** | 10 |

*Mean Side Effects per patient = 1.341
Median Side Effects per patient = 1*
